# Supplementary material for: A Likelihood Approach for Real-Time Calibration of Stochastic Compartmental Epidemic Models
Source: PLoS Comput Biol. 2017 Jan 17;13(1):e1005257. doi: 10.1371/journal.pcbi.1005257 (PMC5240920; doi:10.1371/journal.pcbi.1005257)
Supplement: S1 File — (TAR.GZ) [file pcbi.1005257.s014.tar.gz › HSPH_Online-SI-Revision/EnKF07/n100-mild/n100-mild_EnKF2_table-8.pdf]

| | | | | | | | | | | | | | | | | | | | | | | | | | | | | | | |
|---|---|---|---|---|---|---|---|---|---|---|---|---|---|---|---|---|---|---|---|---|---|---|---|---|---|---|---|---|---|---|
| 1 | newly 3 weeks spec | 1329. | 2359.57 | {0., 4248.} | 222.217 | 1.49395 | newly 3 weeks cumul | 2670. | 7349.47 | {948., 26 803.} | 236.248 | 0.414267 | newly 5 weeks spec | 2409. | 1003.79 | {0., 3086.} | 113.884 | 2.36887 | newly 5 weeks cumul | 6876. | 10 146.9 | {948., 27 446.} | 163.49 | 0.641863 | newly 60 weeks cumul | 33 899. | 11 405.7 | {948., 33 866.} | 91.6367 | 1.10783 |
| 2 | newly 3 weeks spec | 1754. | 1511.7 | {133., 1681.} | 134.404 | 0.815229 | newly 3 weeks cumul | 4109. | 7409.56 | {1307., 10 085.} | 164.259 | 0.379494 | newly 5 weeks spec | 2989. | 543.39 | {13., 1108.} | 95.9415 | 1.57795 | newly 5 weeks cumul | 9407. | 8832.8 | {1465., 11 447.} | 124.047 | 0.613821 | newly 59 weeks cumul | 41 142. | 10 654.9 | {1524., 15 914.} | 94.0177 | 1.10722 |
| 3 | newly 3 weeks spec | 1224. | 1004.64 | {246., 1810.} | 62.9902 | 0.376708 | newly 3 weeks cumul | 2588. | 18 743.4 | {7088., 39 732.} | 624.243 | 0.73159 | newly 5 weeks spec | 2853. | 229.26 | {5., 255.} | 94.4444 | 1.89016 | newly 5 weeks cumul | 7485. | 19 378.9 | {8705., 40 902.} | 161.985 | 0.306938 | newly 62 weeks cumul | 35 853. | 19 878.1 | {8710., 41 023.} | 64.8974 | 0.437353 |
| 4 | newly 3 weeks spec | 1047. | 9787.84 | {1680., 21 114.} | 844.552 | 0.881665 | newly 3 weeks cumul | 1848. | 46 513.7 | {18 122., 85 319.} | 2418.89 | 1.29492 | newly 5 weeks spec | 3916. | 4518.01 | {84., 9807.} | 75.1422 | 0.571695 | newly 5 weeks cumul | 7888. | 57 647.9 | {22 555., 104 267.} | 634.346 | 0.801335 | newly 66 weeks cumul | 49 382. | 70 067.5 | {29 219., 111 766.} | 64.2871 | 0.24054 |
| 5 | newly 3 weeks spec | 1604. | 1190.45 | {252., 2714.} | 69.0592 | 0.430258 | newly 3 weeks cumul | 3273. | 19 650.9 | {8701., 47 394.} | 502.27 | 0.642546 | newly 5 weeks spec | 2839. | 268.28 | {5., 248.} | 94.0486 | 1.78089 | newly 5 weeks cumul | 8315. | 20 401.2 | {8973., 47 428.} | 150.586 | 0.286871 | newly 62 weeks cumul | 37 686. | 20 939.5 | {8983., 47 429.} | 66.5117 | 0.459259 |
| 6 | newly 3 weeks spec | 1697. | 2881.22 | {0., 9319.} | 216.447 | 1.5461 | newly 3 weeks cumul | 3762. | 8963.73 | {936., 44 325.} | 224.264 | 0.535266 | newly 5 weeks spec | 3382. | 1195.75 | {0., 7921.} | 105.603 | 2.37157 | newly 5 weeks cumul | 9658. | 12 308.5 | {936., 63 147.} | 154.575 | 0.777683 | newly 60 weeks cumul | 39 815. | 13 800.6 | {940., 64 948.} | 91.1339 | 1.17836 |
| 7 | newly 3 weeks spec | 621. | 6896.14 | {419., 11 410.} | 1023.02 | 0.928644 | newly 3 weeks cumul | 1186. | 60 813.8 | {23 917., 110 520.} | 5027.64 | 1.6316 | newly 5 weeks spec | 2262. | 1819.27 | {2., 4097.} | 75.7891 | 0.907364 | newly 5 weeks cumul | 4678. | 66 080.4 | {29 087., 119 297.} | 1313.61 | 1.08648 | newly 65 weeks cumul | 38 409. | 68 912.9 | {29 950., 123 789.} | 90.295 | 0.266917 |
| 8 | newly 3 weeks spec | 1914. | 2294.42 | {122., 14 463.} | 161.842 | 0.873228 | newly 3 weeks cumul | 4004. | 10 981.6 | {1346., 48 531.} | 247.282 | 0.414185 | newly 5 weeks spec | 3108. | 643.78 | {12., 2083.} | 92.1165 | 1.58711 | newly 5 weeks cumul | 9610. | 12 793.4 | {1507., 62 172.} | 153.89 | 0.628545 | newly 59 weeks cumul | 33 244. | 14 277.9 | {1556., 66 611.} | 98.8581 | 1.0052 |
| 9 | newly 3 weeks spec | 1331. | 7665.42 | {1290., 20 546.} | 480.084 | 0.624601 | newly 3 weeks cumul | 2488. | 45 965. | {12 421., 96 298.} | 1748.55 | 1.12213 | newly 5 weeks spec | 3831. | 2344.14 | {183., 4360.} | 65.0499 | 0.570039 | newly 5 weeks cumul | 8579. | 52 429.1 | {13 783., 108 219.} | 514.799 | 0.675366 | newly 64 weeks cumul | 48 106. | 56 335.7 | {14 239., 116 781.} | 65.6684 | 0.289545 |
| 10 | newly 3 weeks spec | 2104. | 1050.78 | {83., 1020.} | 89.7605 | 0.745162 | newly 3 weeks cumul | 4740. | 5643.22 | {2276., 5574.} | 76.0658 | 0.23053 | newly 5 weeks spec | 3114. | 543.87 | {0., 595.} | 93.1082 | 1.60466 | newly 5 weeks cumul | 10 319. | 6941.31 | {2704., 5792.} | 80.901 | 0.444625 | newly 58 weeks cumul | 38 689. | 8083.51 | {2965., 6562.} | 85.6288 | 0.895818 |
| 11 | newly 3 weeks spec | 890. | 963.59 | {241., 1824.} | 74.3921 | 0.363069 | newly 3 weeks cumul | 1597. | 20 133.5 | {5861., 67 981.} | 1161.09 | 0.916576 | newly 5 weeks spec | 2552. | 173.87 | {5., 235.} | 94.3774 | 1.85918 | newly 5 weeks cumul | 5799. | 20 637.1 | {7042., 68 348.} | 261.605 | 0.416393 | newly 62 weeks cumul | 44 256. | 21 266.5 | {7045., 68 356.} | 70.8038 | 0.547753 |
| 12 | newly 3 weeks spec | 1981. | 1029.22 | {89., 933.} | 89.0581 | 0.697279 | newly 3 weeks cumul | 4456. | 5581.59 | {2426., 5319.} | 74.4728 | 0.20556 | newly 5 weeks spec | 3330. | 505.38 | {2., 616.} | 93.6 | 1.5711 | newly 5 weeks cumul | 10 451. | 6806.25 | {2869., 5383.} | 79.9073 | 0.442588 | newly 58 weeks cumul | 44 615. | 7904.75 | {3005., 7022.} | 86.8793 | 0.951204 |
| 13 | newly 3 weeks spec | 1116. | 2379.95 | {2., 8113.} | 220.822 | 1.30241 | newly 3 weeks cumul | 2037. | 36 768.3 | {7128., 78 359.} | 1711.99 | 1.11633 | newly 5 weeks spec | 3104. | 773.13 | {0., 3015.} | 80.7368 | 2.51854 | newly 5 weeks cumul | 7098. | 38 815. | {7383., 81 627.} | 458.504 | 0.676215 | newly 63 weeks cumul | 40 666. | 40 775.1 | {7399., 81 969.} | 55.7921 | 0.33823 |
| 14 | newly 3 weeks spec | 1519. | 6794.42 | {315., 11 435.} | 377.264 | 0.677939 | newly 3 weeks cumul | 2463. | 61 597. | {23 939., 121 167.} | 2401.04 | 1.31931 | newly 5 weeks spec | 4569. | 1834.3 | {1., 4105.} | 71.1495 | 1.20576 | newly 5 weeks cumul | 9906. | 66 871.8 | {29 142., 123 637.} | 576.604 | 0.771848 | newly 65 weeks cumul | 48 448. | 69 734.8 | {30 004., 124 443.} | 60.9615 | 0.211521 |
| 15 | newly 3 weeks spec | 1182. | 1325.34 | {267., 2095.} | 87.8545 | 0.392261 | newly 3 weeks cumul | 2013. | 22 186.1 | {6785., 68 182.} | 1002.23 | 0.842609 | newly 5 weeks spec | 3065. | 259.6 | {4., 336.} | 93.3697 | 1.83807 | newly 5 weeks cumul | 7143. | 22 955.1 | {6966., 68 548.} | 226.033 | 0.347276 | newly 62 weeks cumul | 48 233. | 23 664.8 | {7132., 68 559.} | 72.6418 | 0.561528 |
| 16 | newly 3 weeks spec | 1433. | 1076.7 | {102., 1004.} | 95.3901 | 0.592691 | newly 3 weeks cumul | 3454. | 5972.37 | {2430., 5256.} | 94.7623 | 0.170238 | newly 5 weeks spec | 2363. | 518.11 | {2., 607.} | 93.0169 | 1.4466 | newly 5 weeks cumul | 7772. | 7241.82 | {2859., 5851.} | 83.4529 | 0.340478 | newly 58 weeks cumul | 32 590. | 8320.17 | {3012., 6718.} | 84.922 | 0.821307 |
| 17 | newly 3 weeks spec | 933. | 7379.73 | {1277., 20 632.} | 692.284 | 0.747243 | newly 3 weeks cumul | 1663. | 43 833.6 | {12 291., 94 528.} | 2536.36 | 1.27212 | newly 5 weeks spec | 2774. | 2280.22 | {184., 4440.} | 71.5955 | 0.503417 | newly 5 weeks cumul | 6118. | 50 107.8 | {13 928., 108 079.} | 720.76 | 0.788505 | newly 64 weeks cumul | 46 517. | 54 051.2 | {14 270., 112 347.} | 65.7988 | 0.28732 |
| 18 | newly 3 weeks spec | 223. | 5121. | {412., 13 822.} | 2202.19 | 1.10696 | newly 3 weeks cumul | 346. | 36 495. | {4807., 90 961.} | 10 448.1 | 1.75789 | newly 5 weeks spec | 1979. | 3424.36 | {2., 11 804.} | 178.304 | 1.24625 | newly 5 weeks cumul | 3142. | 43 903.4 | {5859., 92 022.} | 1302.89 | 0.974893 | newly 68 weeks cumul | 44 313. | 58 521.4 | {6554., 97 681.} | 80.866 | 0.403751 |
| 19 | newly 3 weeks spec | 1287. | 2450.73 | {131., 14 392.} | 218.52 | 0.769299 | newly 3 weeks cumul | 2744. | 11 738.6 | {1322., 66 198.} | 370.299 | 0.370793 | newly 5 weeks spec | 2837. | 720.79 | {14., 2180.} | 92.4692 | 1.51661 | newly 5 weeks cumul | 7629. | 13 739.8 | {1475., 74 399.} | 189.631 | 0.587526 | newly 59 weeks cumul | 38 174. | 15 761.6 | {1531., 75 538.} | 95.7347 | 1.03538 |
| 20 | newly 3 weeks spec | 216. | 4727.12 | {258., 10 244.} | 2099.47 | 1.10738 | newly 3 weeks cumul | 340. | 38 532.1 | {4896., 94 405.} | 11 233. | 1.80195 | newly 5 weeks spec | 1268. | 3144.64 | {0., 10 066.} | 251.391 | 1.3874 | newly 5 weeks cumul | 2188. | 45 310.3 | {5740., 96 492.} | 1972.83 | 1.12483 | newly 68 weeks cumul | 40 762. | 57 294.2 | {6831., 98 695.} | 89.4997 | 0.407273 |
| 21 | newly 3 weeks spec | 570. | 4744.06 | {244., 13 566.} | 757.049 | 0.816051 | newly 3 weeks cumul | 880. | 34 218.2 | {4478., 90 940.} | 3788.43 | 1.31337 | newly 5 weeks spec | 2390. | 3312.61 | {0., 10 255.} | 149.356 | 1.35533 | newly 5 weeks cumul | 4463. | 41 377.2 | {5573., 91 101.} | 834.153 | 0.793913 | newly 68 weeks cumul | 44 593. | 54 388.1 | {5793., 97 589.} | 78.2744 | 0.419395 |
| 22 | newly 3 weeks spec | 1853. | 1031.56 | {149., 926.} | 89.3654 | 0.661646 | newly 3 weeks cumul | 4544. | 5641.25 | {2422., 5302.} | 73.8642 | 0.206405 | newly 5 weeks spec | 2912. | 505.08 | {5., 593.} | 93.1044 | 1.48471 | newly 5 weeks cumul | 9679. | 6874.45 | {2856., 5438.} | 79.2021 | 0.408887 | newly 58 weeks cumul | 43 141. | 7992.64 | {3006., 6925.} | 86.4881 | 0.930414 |
| 23 | newly 3 weeks spec | 936. | 2485.13 | {3., 8144.} | 269.783 | 1.30194 | newly 3 weeks cumul | 1680. | 38 473.6 | {7176., 85 543.} | 2196.04 | 1.21691 | newly 5 weeks spec | 2751. | 794.6 | {0., 2959.} | 80.5823 | 2.50384 | newly 5 weeks cumul | 5966. | 40 588.4 | {7412., 97 212.} | 589.682 | 0.754945 | newly 63 weeks cumul | 45 926. | 42 579.8 | {7423., 98 225.} | 55.6826 | 0.353945 |
| 24 | newly 3 weeks spec | 678. | 9287.18 | {364., 19 321.} | 1289.13 | 1.0204 | newly 3 weeks cumul | 1172. | 45 134.3 | {10 591., 89 072.} | 3756.13 | 1.4582 | newly 5 weeks spec | 2554. | 5051.76 | {1., 12 392.} | 164.721 | 1.07826 | newly 5 weeks cumul | 4980. | 57 239.1 | {12 575., 97 962.} | 1057.86 | 0.997633 | newly 67 weeks cumul | 42 361. | 68 471.3 | {13 769., 107 448.} | 93.1898 | 0.392399 |
| 25 | newly 3 weeks spec | 1220. | 1075.56 | {98., 887.} | 99.9361 | 0.544455 | newly 3 weeks cumul | 2798. | 6072.27 | {2282., 5347.} | 125.677 | 0.187609 | newly 5 weeks spec | 2397. | 448.43 | {2., 578.} | 90.058 | 1.44607 | newly 5 weeks cumul | 7013. | 7214.88 | {2723., 5417.} | 84.9149 | 0.310822 | newly 58 weeks cumul | 41 718. | 8119.62 | {2997., 6736.} | 86.076 | 0.925425 |
| 26 | newly 3 weeks spec | 1422. | 2474.92 | {0., 4560.} | 218.291 | 1.56098 | newly 3 weeks cumul | 2792. | 7443.4 | {960., 26 839.} | 230.787 | 0.428502 | newly 5 weeks spec | 3228. | 1124.51 | {0., 3981.} | 106.641 | 2.40704 | newly 5 weeks cumul | 8236. | 10 472.2 | {960., 27 537.} | 150.501 | 0.699118 | newly 60 weeks cumul | 43 633. | 12 036.9 | {960., 50 000.} | 89.0517 | 1.1969 |
| 27 | newly 3 weeks spec | 1429. | 1110.86 | {98., 1916.} | 96.662 | 0.602208 | newly 3 weeks cumul | 3015. | 6376.83 | {2341., 6883.} | 122.839 | 0.182344 | newly 5 weeks spec | 2878. | 485.21 | {2., 662.} | 89.73 | 1.49334 | newly 5 weeks cumul | 7889. | 7587.25 | {2807., 9626.} | 85.9515 | 0.344607 | newly 58 weeks cumul | 41 619. | 8576. | {3055., 12 548.} | 85.1522 | 0.904524 |
| 28 | newly 3 weeks spec | 1395. | 1481.4 | {110., 619.} | 150.842 | 0.812389 | newly 3 weeks cumul | 3462. | 7357.62 | {1300., 10 964.} | 185.531 | 0.36074 | newly 5 weeks spec | 2612. | 515.9 | {9., 280.} | 97.758 | 1.64381 | newly 5 weeks cumul | 8023. | 8722.1 | {1454., 11 404.} | 133.657 | 0.59486 | newly 59 weeks cumul | 37 337. | 10 478.6 | {1507., 11 434.} | 95.8298 | 1.1115 |
| 29 | newly 3 weeks spec | 1153. | 396.97 | {0., 542.} | 97.85 | 1.47829 | newly 3 weeks cumul | 2468. | 7732.7 | {836., 18 205.} | 253.02 | 0.442947 | newly 5 weeks spec | 2181. | 126.99 | {0., 89.} | 97.8776 | 2.67593 | newly 5 weeks cumul | 6228. | 8077.11 | {917., 18 352.} | 106.639 | 0.43472 | newly 61 weeks cumul | 30 741. | 8269.88 | {933., 18 352.} | 83.5966 | 0.927684 |
| 30 | newly 3 weeks spec | 1065. | 3113.42 | {2., 8354.} | 291.159 | 1.29841 | newly 3 weeks cumul | 1901. | 40 939.8 | {7194., 91 356.} | 2055.99 | 1.16634 | newly 5 weeks spec | 3590. | 1079.77 | {0., 3137.} | 87.2365 | 2.45626 | newly 5 weeks cumul | 7402. | 43 738. | {7458., 105 471.} | 497.498 | 0.652536 | newly 63 weeks cumul | 47 995. | 46 263.6 | {7476., 116 995.} | 64.1922 | 0.363338 |
| 31 | newly 3 weeks spec | 1303. | 1631.71 | {0., 844.} | 179.167 | 1.59182 | newly 3 weeks cumul | 2596. | 5759.93 | {883., 16 865.} | 193.826 | 0.420934 | newly 5 weeks spec | 2456. | 674.54 | {0., 289.} | 108.61 | 2.47619 | newly 5 weeks cumul | 6882. | 7662.2 | {883., 20 209.} | 137.809 | 0.679726 | newly 60 weeks cumul | 37 472. | 8558.16 | {883., 20 209.} | 90.9436 | 1.23503 |
| 32 | newly 3 weeks spec | 2005. | 1063.79 | {171., 940.} | 90.7087 | 0.709499 | newly 3 weeks cumul | 4624. | 5933.09 | {2285., 5328.} | 80.3026 | 0.220745 | newly 5 weeks spec | 3092. | 506.16 | {6., 615.} | 93.1882 | 1.53105 | newly 5 weeks cumul | 10 135. | 7170.94 | {2736., 5426.} | 82.3964 | 0.433697 | newly 58 weeks cumul | 42 820. | 8248.94 | {2985., 6914.} | 85.8081 | 0.926735 |
| 33 | newly 3 weeks spec | 116. | 5192.57 | {256., 13 762.} | 4383.25 | 1.34035 | newly 3 weeks cumul | 183. | 36 013.5 | {4475., 90 808.} | 19 579.5 | 2.01211 | newly 5 weeks spec | 1068. | 3469.79 | {0., 10 862.} | 320.151 | 1.27694 | newly 5 weeks cumul | 1629. | 43 543.4 | {5576., 90 834.} | 2575.08 | 1.21703 | newly 68 weeks cumul | 33 313. | 56 809. | {5818., 97 580.} | 117.794 | 0.457175 |
| 34 | newly 3 weeks spec | 1093. | 6112.55 | {1194., 10 714.} | 464.349 | 0.632253 | newly 3 weeks cumul | 1962. | 46 037.2 | {11 122., 119 923.} | 2247.4 | 1.20924 | newly 5 weeks spec | 3687. | 1711.37 | {100., 3621.} | 66.2775 | 0.675419 | newly 5 weeks cumul | 7846. | 50 797.6 | {13 893., 123 360.} | 551.713 | 0.694164 | newly 64 weeks cumul | 44 642. | 53 673.5 | {14 361., 123 612.} | 68.0586 | 0.287473 |
| 35 | newly 3 weeks spec | 20. | 4866.45 | {211., 13 121.} | 24 238.5 | 2.08912 | newly 3 weeks cumul | 142. | 37 120. | {4933., 91 935.} | 26 040.8 | 2.17364 | newly 5 weeks spec | 625. | 3046.17 | {0., 10 137.} | 461.134 | 1.21831 | newly 5 weeks cumul | 970. | 43 894.6 | {6037., 92 027.} | 4426.33 | 1.46891 | newly 68 weeks cumul | 40 997. | 56 079.9 | {7221., 98 489.} | 80.5041 | 0.354276 |
| 36 | newly 3 weeks spec | 277. | 4693.61 | {281., 10 454.} | 1606.92 | 1.01912 | newly 3 weeks cumul | 444. | 36 295.5 | {4930., 91 172.} | 8074.66 | 1.65314 | newly 5 weeks spec | 1470. | 3388.01 | {0., 10 275.} | 232.018 | 1.34828 | newly 5 weeks cumul | 2688. | 43 506.2 | {5774., 91 361.} | 1522.48 | 1.02987 | newly 68 weeks cumul | 33 226. | 56 485.8 | {6581., 97 547.} | 112.717 | 0.426912 |
| 37 | newly 3 weeks spec | 1497. | 356.74 | {0., 529.} | 93.8651 | 1.56514 | newly 3 weeks cumul | 2865. | 6778.59 | {1038., 15 167.} | 174.649 | 0.385083 | newly 5 weeks spec | 3432. | 89.12 | {0., 96.} | 97.9994 | 2.88356 | newly 5 weeks cumul | 8555. | 7039.03 | {1055., 15 175.} | 76.5385 | 0.448115 | newly 61 weeks cumul | 44 446. | 7144.9 | {1057., 15 175.} | 86.2989 | 1.04809 |
| 38 | newly 3 weeks spec | 250. | 5163.93 | {359., 14 438.} | 1971.45 | 1.05536 | newly 3 weeks cumul | 547. | 37 667.9 | {4745., 91 891.} | 6787.22 | 1.57137 | newly 5 weeks spec | 1505. | 3315.92 | {3., 11 449.} | 221.272 | 1.15807 | newly 5 weeks cumul | 2547. | 44 921.8 | {5627., 92 019.} | 1668.71 | 1.05899 | newly 68 weeks cumul | 38 637. | 58 432.2 | {6537., 97 731.} | 96.9458 | 0.41806 |
| 39 | newly 3 weeks spec | 1338. | 7326.06 | {702., 15 625.} | 469.277 | 0.673089 | newly 3 weeks cumul | 2487. | 59 146.4 | {27 859., 109 286.} | 2278.72 | 1.30966 | newly 5 weeks spec | 3912. | 2016.81 | {4., 5244.} | 74.7681 | 1.04589 | newly 5 weeks cumul | 8892. | 64 852.6 | {31 214., 115 976.} | 630.847 | 0.811478 | newly 65 weeks cumul | 39 846. | 68 270. | {33 119., 122 645.} | 81.4368 | 0.245452 |
| 40 | newly 3 weeks spec | 586. | 9600.17 | {771., 18 555.} | 1542.47 | 1.01136 | newly 3 weeks cumul | 886. | 55 559.1 | {14 588., 102 215.} | 6170.78 | 1.70867 | newly 5 weeks spec | 3300. | 5364.69 | {2., 12 235.} | 134.775 | 1.09318 | newly 5 weeks cumul | 5752. | 68 154. | {25 337., 104 720.} | 1085.29 | 1.01829 | newly 67 weeks cumul | 45 872. | 81 576.5 | {31 016., 106 936.} | 90.2439 | 0.293807 |
| 41 | newly 3 weeks spec | 397. | 4490.56 | {203., 10 596.} | 1050.88 | 0.90143 | newly 3 weeks cumul | 582. | 34 606.7 | {4527., 90 952.} | 5846.16 | 1.4995 | newly 5 weeks spec | 2106. | 3327.72 | {0., 10 058.} | 171.366 | 1.42698 | newly 5 weeks cumul | 3564. | 41 630.2 | {5726., 90 997.} | 1073.49 | 0.886502 | newly 68 weeks cumul | 48 915. | 55 165.4 | {5779., 97 567.} | 70.2086 | 0.407623 |
| 42 | newly 3 weeks spec | 795. | 6322.19 | {291., 11 179.} | 716.26 | 0.779836 | newly 3 weeks cumul | 1426. | 61 018. | {23 970., 110 514.} | 4180.36 | 1.51679 | newly 5 weeks spec | 2995. | 1530.11 | {2., 4038.} | 71.1035 | 1.06836 | newly 5 weeks cumul | 5937. | 65 557. | {25 426., 117 262.} | 1008.45 | 0.972903 | newly 65 weeks cumul | 44 109. | 68 425.6 | {32 723., 123 814.} | 72.8274 | 0.256362 |
| 43 | newly 3 weeks spec | 339. | 5052.53 | {291., 13 324.} | 1400.65 | 0.926904 | newly 3 weeks cumul | 542. | 33 708.5 | {4834., 88 605.} | 6120.15 | 1.53983 | newly 5 weeks spec | 1978. | 3455.9 | {1., 10 913.} | 180.039 | 1.26362 | newly 5 weeks cumul | 3344. | 41 201.9 | {5847., 90 190.} | 1136.84 | 0.91925 | newly 68 weeks cumul | 47 323. | 54 334.3 | {6752., 97 560.} | 71.2597 | 0.399542 |
| 44 | newly 3 weeks spec | 386. | 9221.97 | {355., 18 868.} | 2303.29 | 1.19329 | newly 3 weeks cumul | 600. | 45 550.5 | {10 196., 91 898.} | 7496.08 | 1.7247 | newly 5 weeks spec | 2455. | 4998.52 | {1., 12 381.} | 171.133 | 1.11119 | newly 5 weeks cumul | 4090. | 57 545. | {12 523., 97 992.} | 1315.96 | 1.07815 | newly 67 weeks cumul | 47 913. | 68 813.9 | {13 799., 107 377.} | 76.0075 | 0.367152 |
| 45 | newly 3 weeks spec | 1342. | 2347.32 | {1., 8083.} | 181.848 | 1.30584 | newly 3 weeks cumul | 2662. | 35 864.8 | {4987., 74 608.} | 1253.96 | 0.990734 | newly 5 weeks spec | 2863. | 752.59 | {0., 2868.} | 89.9738 | 2.51772 | newly 5 weeks cumul | 7410. | 37 847.6 | {7123., 74 608.} | 421.772 | 0.633094 | newly 63 weeks cumul | 42 303. | 39 483.3 | {7461., 91 445.} | 58.655 | 0.364706 |
| 46 | newly 3 weeks spec | 594. | 6152.42 | {1093., 13 799.} | 938.256 | 0.880505 | newly 3 weeks cumul | 1181. | 44 811.6 | {13 429., 94 143.} | 3694.38 | 1.4519 | newly 5 weeks spec | 2667. | 1602.57 | {85., 3688.} | 67.7057 | 0.635014 | newly 5 weeks cumul | 5207. | 49 454.3 | {14 092., 104 649.} | 851.226 | 0.873641 | newly 64 weeks cumul | 48 956. | 51 792.1 | {14 948., 106 956.} | 57.2204 | 0.268229 |
| 47 | newly 3 weeks spec | 763. | 5799.45 | {1106., 11 880.} | 661.419 | 0.741 | newly 3 weeks cumul | 1478. | 40 112.8 | {12 215., 92 469.} | 2614.46 | 1.29634 | newly 5 weeks spec | 3193. | 1647.04 | {126., 3537.} | 65.1788 | 0.637436 | newly 5 weeks cumul | 6436. | 44 738.7 | {14 076., 96 286.} | 598.401 | 0.737241 | newly 64 weeks cumul | 48 321. | 47 367.2 | {14 216., 99 259.} | 54.5513 | 0.275965 |
| 48 | newly 3 weeks spec | 1223. | 2457.41 | {147., 14 635.} | 226.065 | 0.694319 | newly 3 weeks cumul | 2426. | 11 251.1 | {1331., 65 883.} | 391.493 | 0.312573 | newly 5 weeks spec | 3101. | 520.89 | {18., 2165.} | 88.9252 | 1.44905 | newly 5 weeks cumul | 7512. | 12 896.7 | {1488., 74 058.} | 183.854 | 0.561571 | newly 59 weeks cumul | 44 170. | 13 683.4 | {1535., 75 307.} | 92.0718 | 1.09574 |
| 49 | newly 3 weeks spec | 820. | 6734.91 | {311., 11 465.} | 739.63 | 0.840469 | newly 3 weeks cumul | 1602. | 62 428.6 | {23 987., 121 014.} | 3796.91 | 1.51016 | newly 5 weeks spec | 2523. | 1775.36 | {0., 4016.} | 74.6556 | 1.05875 | newly 5 weeks cumul | 5593. | 67 573.6 | {29 087., 123 445.} | 1109.4 | 1.01875 | newly 65 weeks cumul | 35 659. | 70 350.4 | {30 062., 124 516.} | 106.747 | 0.297783 |
| 50 | newly 3 weeks spec | 648. | 8835.9 | {1617., 20 857.} | 1274.27 | 1.04035 | newly 3 weeks cumul | 1146. | 40 765.6 | {16 381., 75 353.} | 3458.52 | 1.43152 | newly 5 weeks spec | 2869. | 4270.94 | {200., 8893.} | 93.2137 | 0.583976 | newly 5 weeks cumul | 5616. | 51 219.7 | {21 966., 96 213.} | 816.083 | 0.892065 | newly 66 weeks cumul | 40 068. | 62 651.2 | {27 484., 105 257.} | 73.0886 | 0.250064 |
